# Efficient musculoskeletal annotation using free-form deformation

**DOI:** 10.1038/s41598-024-67125-3

**Published:** 2024-07-12

**Authors:** Norio Fukuda, Shoji Konda, Jun Umehara, Masaya Hirashima

**Affiliations:** 1 Center for Information and Neural Networks (CiNet), Advanced ICT Research Institute, National Institute of Information and Communications Technology (NICT), 1-4 Yamadaoka, Suita, Osaka 565-0871 Japan; 2 Department of Health and Sport Sciences, Graduate School of Medicine, Osaka University, 1-17 Machikaneyama-Cho, Toyonaka, Osaka 560-0043 Japan; 3 Faculty of Rehabilitation, Kansai Medical University, 18-89 Uyama-Higashi, Hirakata, Osaka 573-1136 Japan; 4 Graduate School of Frontier Biosciences, Osaka University, 1-3 Yamadaoka, Suita, Osaka 565-0871 Japan

**Keywords:** Dataset creation, Deep learning, Free-form deformation, Medical image, Muscle segmentation, Non-expert, Medical imaging, Musculoskeletal system, Biomedical engineering

## Abstract

Traditionally, constructing training datasets for automatic muscle segmentation from medical images involved skilled operators, leading to high labor costs and limited scalability. To address this issue, we developed a tool that enables efficient annotation by non-experts and assessed its effectiveness for training an automatic segmentation network. Our system allows users to deform a template three-dimensional (3D) anatomical model to fit a target magnetic-resonance image using free-form deformation with independent control points for axial, sagittal, and coronal directions. This method simplifies the annotation process by allowing non-experts to intuitively adjust the model, enabling simultaneous annotation of all muscles in the template. We evaluated the quality of the tool-assisted segmentation performed by non-experts, which achieved a Dice coefficient greater than 0.75 compared to expert segmentation, without significant errors such as mislabeling adjacent muscles or omitting musculature. An automatic segmentation network trained with datasets created using this tool demonstrated performance comparable to or superior to that of networks trained with expert-generated datasets. This innovative tool significantly reduces the time and labor costs associated with dataset creation for automatic muscle segmentation, potentially revolutionizing medical image annotation and accelerating the development of deep learning-based segmentation networks in various clinical applications.

## Introduction

Because the volume of muscle is an important determinant of its force-producing capacity, quantification of individual muscle volume in vivo is of considerable interest in the fields of health, medicine, and sports^1,2^. Muscle volume or cross-sectional area has been assessed by segmenting muscles using magnetic-resonance (MR) images^1–6^ or computed-tomography images^7–9^. Segmentation is typically performed manually by experts. However, manual segmentation (the task of tracing the contour of each muscle slice-by-slice) is time-consuming–particularly when the number of muscles is large.

Recently, automatic segmentation techniques based on deep neural networks and artificial intelligence (AI) have been revolutionized^10–12^. These techniques have been applied to muscle segmentation: once the network is trained with a dataset of several dozen cases, it can instantaneously recognize individual muscles from medical images with similar accuracy to an expert (Dice coefficient of 0.8 – 0.9)^13–21^. Such AI-based methods are promising; however, to train a network for different body parts or subject groups, manual segmentation or annotation tasks are necessary to create a training dataset. Thus, there is a growing need for techniques that assist in the annotation and efficient creation of datasets.

Several annotation-assistance methods, including classical techniques and recently developed AI-assisted methods, have been proposed for image processing. Classical techniques such as “graph cuts” and “snakes” help operators to trace the boundaries of objects of interest by utilizing the local intensity features of the image^22,23^. However, these approaches often struggle to effectively annotate structures with unclear boundaries, such as muscles. In contrast, AI-assisted techniques provide an interactive framework where AI iteratively suggests segmentations based on expert corrections through simple scribbling or clicking, continuing until the expert is satisfied^24–26^. This increases the efficiency of the annotation process but typically requires substantial pre-training and may not provide satisfactory results unless the operational conditions of the trained model (such as imaging modality and acquisition area) are satisfied.

In the aforementioned studies, researchers assumed that datasets should be created according to expert judgment. However, the reliance on experts inevitably incurs time and human-resource costs. Therefore, in the present study, we developed a system that allows segmentation even by non-experts. A key innovation is allowing non-experts to annotate muscles by deforming a template 3D anatomical model that includes full musculoskeletal elements to fit the target MR image. If the template model can be deformed while maintaining the topology of internal structures, it can serve as an alternative to the anatomical knowledge typically provided by experts.

The manipulation of a complex object shape has been performed with free-form deformation (FFD)^27^, which is a powerful modeling tool for describing deformation by manipulating the locations of the control points. FFD manipulation is implemented in several modeling software programs, where users can intuitively deform an object (2D image or 3D surface data) by dragging control points with a mouse.

**Figure 1:**
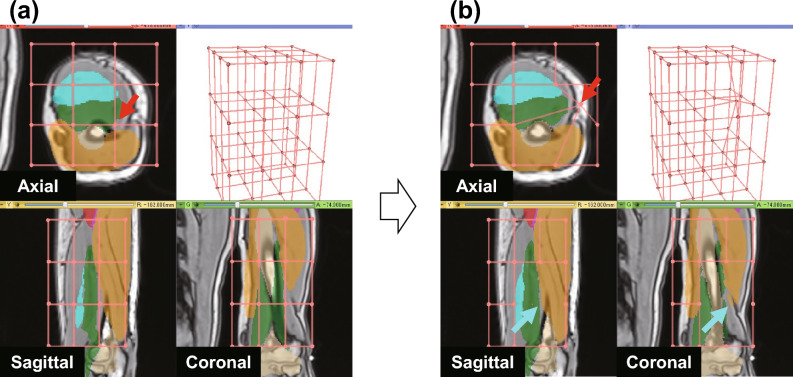
Difficulty in FFD with one set of control points. (**a**) One set of control points is shared for axial, sagittal, and coronal views. (**b**) If a user moves a control point in a certain plane (red arrow), that point disappears from the other plane (cyan arrows).

Although a single set of control points is normally established in FFD, handling these control points across three cross-sectional (axial, coronal, and sagittal) interfaces, which are commonly used for reading 3D medical images, is challenging (see Fig. 1). For example, when a user moves a control point on an axial plane (as indicated by the red arrows in Fig. 1a,b), the moved control point disappears from the planes in the other directions (as indicated by the cyan arrows on the coronal and sagittal planes in Fig. 1b), where it was originally located, making it difficult to track the control point.

**Figure 2:**
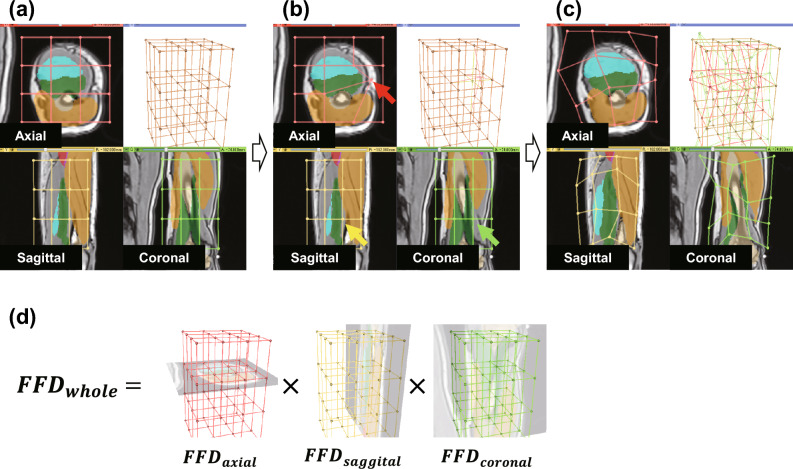
FFD based on three independent sets of control points. (**a**) Three different sets of control points are set for the template model. (**b**) Manipulating a control point on a certain plane (red arrow) does not affect the control points associated with other planes (yellow and green arrows). (**c**) Users can deform the template from the other planes also (yellow and green) without losing sight of the control points. (**d**) Whole deformation field is calculated by multiplying the three deformation fields.

We address this challenge by creating three independent sets of control points corresponding to the axial (red), sagittal (yellow), and coronal (green) directions (Fig. 2d). The entire deformation field is calculated by multiplying the three deformation fields. With respect to a specific direction (e.g., axial), only the corresponding set of control points (e.g., red control points) is displayed and can be manipulated within that plane (Fig. 2a). In this setting, manipulating a control point on a certain plane (indicated by the red arrow in Fig. 2b) does not affect the control points associated with other planes (indicated by the yellow and green arrows in Fig. 2b). This approach allows the intricate deformation of the template through manipulation across all three planes (as demonstrated by the red, yellow, and green grids in Fig. 2c) without losing track of the control points.

The template model can be deformed by the entire deformation field, presented interactively to the user, and superimposed on the target MR image. The user is asked to deform the model by manipulating the control points to ensure that the model accurately fits the MR images. Although it is unrealistic to expect non-experts without anatomical knowledge to manually segment each muscle individually, graphically fitting the template model to the target MR image can be intuitively accomplished, even by non-experts. Importantly, once this task is completed, annotation of all the muscles in the template model is simultaneously achieved, making the process highly efficient.

In this study, we conducted several key tasks to ensure the effectiveness of our system. First, non-experts without specialized anatomical knowledge were engaged in segmentation of dozens of MR image data, and the system’s effectiveness was verified. Second, we trained a deep neural network using the dataset obtained in this study and assessed its ability to perform automatic segmentation. Furthermore, we compared the performance of this network with that of a network traditionally trained using a dataset annotated by individuals with anatomical knowledge. The objective of these investigations was to demonstrate how our system can streamline the creation of training datasets for AI-based automatic segmentation, optimizing both the time-efficiency and cost-effectiveness of the annotation process.

## Materials and methods

### Image data collection

We collected MR images of the upper-extremity musculoskeletal system from 88 healthy male participants (age 22.7 ± 2.6 years, height 172.0 ± 5.2 cm, weight 64.0 ± 9.1 kg). The experiment was approved by the Ethics Committee of the National Institute of Information and Communications Technology (Approval Number: B160051702, Approval Date: January 18, 2018) and was conducted in accordance with the Declaration of Helsinki. All the participants provided written informed consent before participating in the study.

The imaging protocol utilized a 3-T MR system (MAGNETOM Vida, Siemens Healthineers) with a T1-weighted VIBE (Volumetric Interpolated Breath-hold Examination) Dixon sequence (acquisition plane: coronal; field of view: 448 mm × 448 mm; slice thickness: 1.5 mm; resolution: 1.0 mm × 1.0 mm × 1.5 mm; repetition time: 5.88 ms, echo time: 2.46 ms for in-phase image and 1.23 ms for opposed-phase image; flip angle: 5°). The scanning sequence was repeated three or four times by moving the bed from the neck to the wrist to cover the target region of the left upper limb. To reduce the distortion around the boundary of the field of view, the center of the image was moved by 196 or 248 mm with image overlaps of 250 or 200 mm, respectively. Three or four images were reconstructed as having a large field of view using the composing software installed in the console application (Syngo MR XA10, Siemens Healthineers). Opposed-phase images were used in this study because they provide better visibility of the anatomical structures around the muscle attachment area.

### Template model

**Figure 3:**
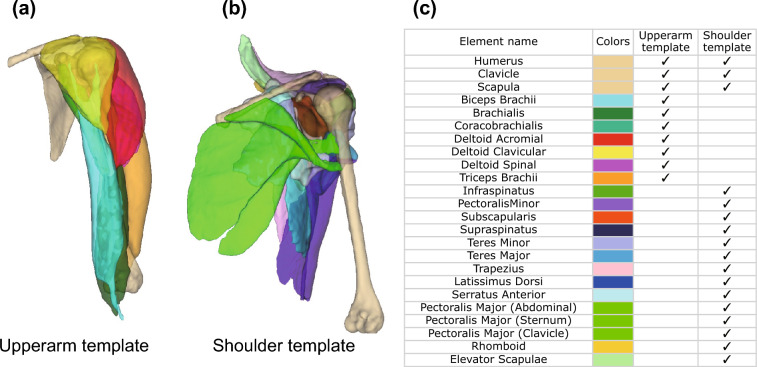
Template model for the upper-extremity musculoskeletal system: (**a**) left upper arm; (**b**) left shoulder; (**c**) name and color of each element.

We devised a novel segmentation support tool that utilizes the FFD of a template human musculoskeletal model. Two template models were used in this study: one for the left upper arm (Fig. 3a) and another for the left shoulder (Fig. 3b). Fig. 3c presents the 24 elements comprising the template models. The models were created by an expert who manually segmented the muscles and bones from a participant’s MR image in a conventional manner using the 3D Slicer platform^28^ over a period of >50 h. They comprised 1-mm-grid 3D volume data, with each voxel having one of the 25 labels (24 elements and background).

### Deformation of template model

Our system overlays the template model and the MR image to be segmented in each view of the three cross-sectional (axial, coronal, and sagittal) interfaces (Fig. 2). To allow the user to manipulate the control points without losing sight of them, we introduced three sets of control points (red, yellow, and green) corresponding to the axial, sagittal, and coronal directions, respectively (Fig. 2). Each set of control points (e.g., red points) is visible only in view of the corresponding plane (e.g., axial) and is allowed to move within the plane. This constraint ensures that all the control points are on the prescribed planes, preventing users from losing sight. The system interactively displays deformations in response to the user actions conducted thus far in all three directions. During this process, the template model is deformed by the whole deformation field ($FF{D}_{whole}$), which is calculated by multiplying the three individual deformation fields (see Fig. 2d). Each field is determined by the current locations of the control points in each direction. More specifically, deformation of the template was performed in the following manner. First, the coronal deformation field ($FF{D}_{coronal}$) was applied to the template shape. Next, the sagittal deformation field ($FF{D}_{sagittal}$) was applied to the resulting shape. Finally, the axial deformation field ($FF{D}_{axial}$) was applied to the resulting shape. Changing the order during the segmentation task could cause unintended deformations. However, this issue did not occur in our study as we consistently applied the deformations in the same order.

### Implementation and workflow

We developed a segmentation tool (Seg Muscle) as a plugin for the widely used 3D Slicer platform^28^—a powerful medical image processing software program. Thus, users can leverage the extensive functionality of 3D Slicer while benefiting from the unique features of our plugin. The typical workflow for the segmentation task using our plugin in 3D Slicer is as follows (see also the video on the Seg Muscle website described in the Appendix):

1. The user loads the target medical image into the 3D Slicer.
2. The user selects our plugin, chooses the appropriate template anatomical model, and then performs the initial alignment over the target image using intuitive mouse operations.
3. The user manipulates the control points on the template model to fit the target image.
4. As the user adjusts the control points, the software updates the shape of the template model in real time, providing immediate feedback on the quality of the fit.
5. Once satisfied with the fit, the user saves the results, and the process is complete. If not satisfied, the user can employ the current deformation result as the initial value and then finely adjust the fit using denser control points in the subsequent step.

### Tool-assisted segmentation

Non-experts performed segmentation tasks on the data from 87 participants using our tool. These non-experts were four individuals experienced in using general office applications on a personal computer but with limited anatomical knowledge.

They were instructed to initially fit the template model in a certain direction (e.g., axial), then adjust it in the next direction (e.g., coronal), and finally adjust it in the remaining direction (e.g., sagittal). However, it is important to note that they were not instructed to adjust all the control points in a slice, nor were they instructed to perform the task in every slice. However, they were instructed to perform the task in all three directions for at least two rounds for each participant’s data. Deforming the template model while preserving its topology has the advantage of allowing non-experts to effectively perform segmentation. However, fine-tuning each muscle in our system is difficult because manipulating a control point simultaneously changes the shape of multiple muscles. Therefore, to avoid wasting time, we informed the operators that fine-tuning the individual muscles was not necessary. No time restrictions were imposed. On average, segmentation took approximately 2 h per participant, with the shortest time being approximately 30 min and the longest approximately 4 h.

### Refinement of segmentation

We also created conventional manual segmentation data. These data were created by medical students, physical therapists, or equivalents with a basic understanding of musculoskeletal anatomy and experience in reading medical images. This process refined the results of the tool-assisted segmentation described above. They manually modified the boundary of each muscle individually using the 3D Slicer platform, without our plugin. They were instructed to complete the task within 1 h per element. On average, about 20 hours were spent per participant’s data. After this process, an expert with approximately eight years of experience in manually segmenting muscles from medical images reviewed and further adjusted the data. In total, data for 20 participants were created in accordance with a previous study in which a deep neural network with a dataset of 20 participants exhibited good classification performance^14^.

### Dataset for network training

The dataset used for training the network consisted of MR image data and corresponding label data. The MR image data comprised 400 – 450 axial images per participant, each having dimensions of 448 mm × 264 mm, with a 1-mm gap. These images covered all the elements listed in Fig. 3c. The label data consisted of 1-mm-grid 3D volume data, where each voxel was assigned one of 23 labels (22 elements and background), in which the three parts of the pectoralis major were combined. The following three datasets were used.

1. Expert–20: This is a conventional manual segmentation dataset consisting of the refined label data of 20 participants. It served as the ground truth because of the involvement of an expert in the process.
2. Tool–20: This dataset consists of tool-assisted segmentation labels for the same 20 participants as the Expert–20 dataset. It allowed comparison between the manual and tool-assisted segmentation methods for the same set of participants.
3. Tool–87: The third dataset consisted of tool-assisted segmentation labels for all 87 participants. This dataset provided insights into the effects of applying the tool-assisted method to a larger number of participants.

### Automated segmentation network

We employed a 2D U-Net with an added dice loss function, which is widely used in deep learning^29,30^. This widely recognized and standardized architecture was chosen because it allows us to focus on the performance differences attributable to the quality of the datasets (Expert-20, Tool-20, and Tool-87). Additionally, it would make it easier for other researchers to interpret the results and reproduce the experiments. For implementation, we used the PyTorch framework. In addition, we applied data augmentation during training using the albumentation library.

The training parameters were as follows: a learning rate of 0.0001, the Adam optimization algorithm, a batch size of 8, 30 epochs, mixed-precision training enabled, learning rate scheduling with ReduceLROnPlateau, and early stopping if there was no improvement in the validation metric for five consecutive evaluations conducted 10 times per epoch across epochs. The experiments were performed on a computer with the following specifications: CPU, Intel(R) Xeon(R) E5-2698 v4 @ 2.20 GHz; GPU, Tesla V100 DGXS 32 GB; RAM, 256 GB; OS, Ubuntu 18.04.

We trained the network using one of three datasets to obtain a classifier capable of classifying every voxel into 23 labels (22 elements and background) for a given set of MR image data. To evaluate the performance of the classifier, we employed fivefold validation. For the Expert-20 and Tool–20 datasets, the classifier was trained using data from 16 participants in each fold. The classification results for the data of the remaining four participants in each fold were compared with the ground truth. The procedure for the Tool–87 dataset was similar to that for the Tool–20 dataset. However, for the Tool–87 dataset, the classifier was trained using the data from 83 participants in each fold. This included the same 16 participants as the Tool–20 dataset and 67 additional participants. The agreement between the classification results and ground truth was quantified using the Dice coefficient.

### Declaration of Generative AI and AI-assisted technologies in the writing process

During the preparation of this manuscript, we used the GPT-4 model developed by OpenAI to improve readability and language. After using this tool, the authors reviewed and edited the content as required. We take full responsibility for the content of the manuscript.

## Results

### Quality of tool-assisted segmentation

**Figure 4:**
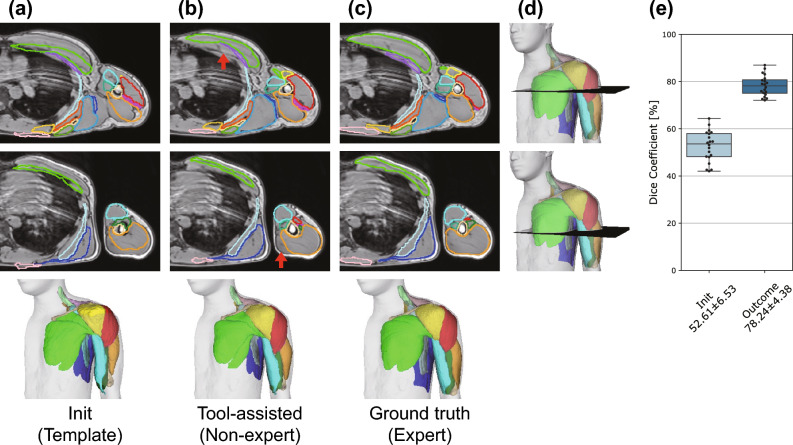
Results of tool-assisted segmentation. Representative axial cross-sectional images with (**a**) initial template labels, (**b**) tool-assisted labels, and (**c**) ground-truth labels for the same MR image. (**d**) Locations of the axial cross-sectional images. (**e**) Dice coefficient comparing initial template labels and tool-assisted labels with ground-truth labels. The third row in (**a**), (**b**), and (**c**) displays the corresponding entire 3D view of the segmentation examples.

Fig. 4 shows representative axial cross-sectional images comparing the initial template labels (Fig. 4a), tool-assisted labels from non-experts (Fig. 4b), and ground-truth labels from experts (Fig. 4c) for the same participant data. The initial template labels (Fig. 4a) indicate a state in which only the location is roughly aligned with the MR image. Because no deformation was performed yet, the agreement between the initial template labels and ground-truth labels was poor; quantitative analysis using 20 participants’ data revealed that Dice coefficient was 52.6% ± 6.53% (Fig. 4e). Fig. 4b shows the state after segmentation by a non-expert using our tool. Importantly, tool-assisted labels do not include beginners’ mistakes, such as adjacent muscles being labeled as opposite to each other, which is attributed to the tool’s ability to deform the template while maintaining the topology. In the visual inspection, the tool-assisted labels (Fig. 4b) exhibited good agreement with the ground-truth labels (Fig. 4c) for most of the elements, although there were mismatches for a few muscles, e.g., the pectoralis minor (red arrow in the upper panel of Fig. 4b) and the medial part of the triceps brachii (red arrow in the lower panel of Fig. 4b). Quantitative analysis of data from 20 participants indicated that the Dice coefficient increased to 78.2% ± 4.38% with tool-assisted segmentation by non-experts (Fig. 4e).

### Network training and classification result

**Figure 5:**
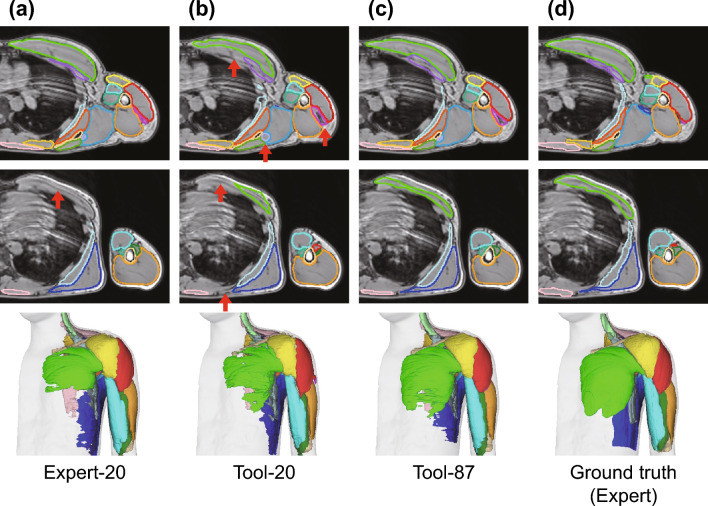
Segmentation results for three classifiers. Representative axial cross-sectional images comparing the classification results for the (**a**) Expert–20, (**b**) Tool–20, and (**c**) Tool–87 datasets and (**d**) the ground truth for the same MR image. The third row in (**a**), (**b**), (**c**), and (**d**) displays the corresponding entire 3D view of the segmentation examples.

The network was trained using each of the three datasets to obtain the classifier. The training time for N = 20 and N = 87 datasets was approximately 1.5 and 9 h, respectively. The obtained classifier could classify muscles and bones from the MR images of one participant within approximately 30–40 s (35 ms per slice), excluding file loading. Fig. 5 shows representative classification results for each classifier. Consistent with previous studies, the classifier trained with the Expert–20 dataset exhibited good performance (compare Fig. 5a,d), although there was an underestimation for a few muscles (see the red arrow in Fig. 5a). The classifier trained with the Tool–20 dataset exhibited discrepancies for several muscles (red arrows in Fig. 5b), likely owing to its relatively coarse segmentation compared with the Expert–20 dataset. Interestingly, training with a larger dataset (i.e., Tool–87) mitigated these discrepancies (see Fig. 5c).

**Figure 6:**
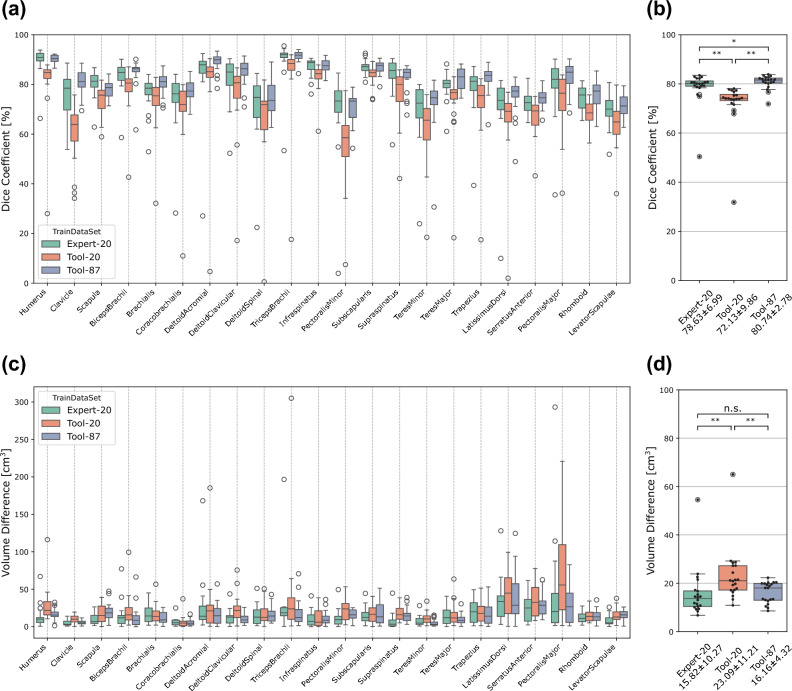
Classification performance for the Expert–20, Tool–20, and Tool–87 classifiers. (**a**) Dice coefficient between the classification result and the ground truth for the 22 musculoskeletal elements averaged among the 20 participants’ data. (**b**) Each dot corresponds to individual participant data averaged over 22 elements. Boxes denote the 1st and 3rd quartiles, and the median is marked with a horizontal line in each box. (**c**) Volume error between the classification results and the ground truth for the 22 musculoskeletal elements averaged among the 20 participants’ data. (**d**) Box plots of the volume error averaged over 22 elements.

Figure 6 quantitatively illustrates the differences in classification performance among the three classifiers. Figure 6a shows the Dice coefficient between the classification results and ground truth for the 22 musculoskeletal elements averaged from the data of 20 participants. Although there were differences in the Dice coefficient among different muscles, it was consistently smaller for the Tool–20 classifier than for the Expert–20 classifier; however, it increased from Tool–20 to Tool–87. The data averaged over 22 elements are presented as box plots in Fig. 6b. The average and standard deviation of the Dice coefficient for the Expert–20, Tool–20, and Tool–87 classifiers were 78.6% ± 6.99%, 72.1% ± 9.86%, and 80.7% ± 2.78%, respectively. Wilcoxon signed-rank tests with Bonferroni correction indicated that the Dice coefficient for the Tool–20 classifier was significantly smaller than that for the Expert-20 classifier (Z = 3.92, corrected P < 0.001) and that it increased from Tool–20 to Tool–87 (Z = 3.92, corrected P < 0.001), surpassing that of Expert–20 (Z = 2.54, corrected P = 0.033). In addition to the Dice coefficient, we also analyzed the volume error for each classifier (Fig. 6c,d). The average and standard deviation of the volume error for the Expert-20, Tool-20, and Tool-87 classifiers were 15.82 ± 10.27 cm^3^, 23.09 ± 11.21 cm^3^, and 16.16 ± 4.32 cm^3^, respectively. Wilcoxon signed-rank tests with Bonferroni correction indicated that the volume error for the Tool–20 classifier was significantly larger than that for the Expert-20 classifier (Z = 3.92, corrected P < 0.001) and that it decreased from Tool–20 to Tool–87 (Z = 3.43, corrected P < 0.01), which is comparable to that of Expert–20 (Z = 1.42, corrected P = 0.468).

## Discussion

### Effectiveness of the proposed tool

Our study highlights the effectiveness of the proposed tool in allowing non-experts to perform segmentation. It is important to note that non-experts can rapidly generate accurate labels without conspicuous errors such as mislabeling of adjacent muscles or omission of musculature. Although the segmentation quality was relatively coarse compared with the precision achieved by experts, the quality (Dice coefficient of approximately 78%) reached a level similar to that of the network trained with Expert–20. Asking non-experts to manually segment each muscle individually is unrealistic; however, our results suggest that they can effectively fit the template model to the target MR image graphically, which serves as a form of segmentation. Another advantage of this template-model approach is that once the deformation is complete, the segmentation of all the muscles is accomplished simultaneously. Typically, the time required for manual segmentation is proportional to the number of elements involved; however, with the proposed tool, the number of muscles ceases to be a limiting factor. Consequently, our tool reduces labor costs and enhances scalability.

### Effect of training data accuracy and quantity on classification performance

Although the proposed tool has many advantages, it is not without limitations. Because the design is aimed at preserving the topology of the template, the generated labels may not perfectly capture the detailed shape of an individual. In fact, there was a tendency for more under- or over-segmentation for the tool-assisted labels than for the ground-truth labels. Accordingly, when using an equivalent amount of participant data, the Tool–20 classifier exhibited significantly inferior performance to the Expert–20 classifier. However, as demonstrated by the Tool–87 classifier, increasing the number of participants can increase the accuracy beyond that of the Expert–20 classifier. This outcome implies that with regard to network performance, accumulating more data can compensate for issues in data quality. Of course, gathering a large amount of high-quality data is ideal. However, considering that this is expensive and time-consuming, our findings suggest that there is also a viable strategy for acquiring a large amount of data that can be easily collected, even if the quality of the data is slightly inferior. Although it is an estimate, Expert-20 required >400 h of expert time, whereas Tool–87 needed approximately 178 h of non-expert time. This indicates that tool-assisted segmentation is a viable option.

However, there is no assurance that gathering large amounts of relatively coarse data will always be beneficial. If the errors in the coarse dataset are biased in a certain direction relative to the true values, the network trained with this dataset could mistakenly recognize the muscle in that direction. Thus, the increased accuracy observed in this study suggests that the errors in tool-assisted labels are uniformly distributed around the true value. We suspect that this uniform error distribution may be due to the properties implicitly inherent in the tool. In our tool, control points are placed coarsely relative to the individual muscles (with the finest grid being 20 mm), and moving a control point deforms not only the current target muscle but also multiple surrounding muscles, which may cause unintentional (and thus unbiased) errors for surrounding regions. Furthermore, in the initial alignment of the template model on the target image (Fig. 4a), there should be no specific bias in the relationship between the initial control points and the individual muscles in the image since the user only roughly aligns the entire template model with the target image. This may further unbias the aforementioned unintentional errors. However, this is only a hypothesis and requires further investigation. How to quantify the error distribution is not a trivial question because muscle boundaries are located everywhere and in every direction. In future studies, we will have to first establish methods to quantify error distribution by focusing on specific boundaries and aim to provide definitive guidelines and bias detection mechanisms. Additionally, it would be desirable to develop a system that detects bias and alerts the users before training AI models.

### Opportunities for the tool usage

Although networks for automatic muscle segmentation have been developed, an important consideration is the need for a segmentation dataset of body parts. To model the entire body, segmentation data for muscles throughout the body are essential. To date, most studies have focused on the lower limbs^13–17^, and a few studies have focused on the upper limb^18–20^ and trunk^21^. Thus, to realize the automatic recognition of the entire body, we still need to create a segmentation dataset for these parts.

Additionally, the accuracy of recognition can be influenced by not only the body part but also the attributes of the individual, such as age, sex, and pathology^18,31^. Hence, to build a universal network, a dataset encompassing a wide range of attributes is necessary, whereas to build a specialized network targeting specific groups, datasets tailored to these attributes are needed. Therefore, manual segmentation remains a critical task in many instances, for which our tool can be effectively utilized.

### Applicability of the template

As this study only examined healthy young males, it is necessary to consider whether our template is applicable to populations with different anatomical presentations, such as older people or patients with some musculoskeletal diseases. Since our tool deforms the template while keeping the topology of the elements, the healthy template model is not directly applicable to such data. However, since the overall topology is preserved, it may be possible to apply it to these data, even for older people or patients, by modifying the template model for each population. For instance, in older subjects, it would be necessary to add fat elements under the skin or between muscles. On the other hand, some muscles may no longer be visible in some slices for patients who have had hip replacement surgery. In such cases, the template requires modification, but most of the region can still be used as is. However, determining effective templates for each population is a subject for future research.

Next, we will discuss the tool’s applicability to different postures like crossing the arms over the body. Although, ideally, the posture between the template model and the body in the image should match, the applicability of the standard template is not compromised by differences in posture. This is because our tool performs segmentation not only of muscles but also of bones using free-form deformation, meaning that adjustments in posture are resolved simultaneously with the deformation of muscles. Thus, as long as the topology does not change significantly, some degree of adaptability is possible. However, if there are significant differences in posture (e.g., shoulder abduction at 0 deg. vs. 90 deg.), significant adjustment is required in the tool segmentation process, which increases the workload substantially. In such cases, it would be more practical to prepare a new template.

### Modality-free approach

An AI-assisted segmentation tool is also available for dataset creation^24–26^. With the assistance of AI, users can interactively perform segmentation using simple operations. However, the AI must be trained on the modalities and body parts of the data being analyzed. In contrast, our tool offers a modality-free approach for both the template model and segmentation target image. As the template model is merely labeled volume data, where each voxel is assigned one of the musculoskeletal element labels, it can be created from any image source. For instance, a template can be generated from a specialized source such as the Visible Korean Human dataset^32^, which contains high-resolution and specialized images. Once created, regardless of the modality used in its development, a template can be used in our tool to segment various 3D medical images, including MRI, CT, and 3D-US, further broadening the applicability of the tool. Although we used templates created from MR images to segment the MR images in this study, our tool is flexible, allowing it to accommodate a wide range of medical imaging scenarios and requirements. Additionally, it is important to highlight that our tool can be used to analyze image data of any resolution. Segmenting muscles from low-resolution data without this tool can be extremely challenging, but by fitting a high-resolution template model to the low-resolution images, it is possible to easily obtain high-resolution segmentation results.

Considering that the modality-free nature of this tool significantly broadens its range of applications and enhances its value to the medical and image-processing communities, we have provided the tool as a plugin for 3D Slicer. It can be used free of charge for academic or non-commercial purposes (see the Appendix). Moreover, we believe that this tool can be an aid in understanding anatomy, making it useful not only as a segmentation tool but potentially also as an educational resource for medical students.

### Future research directions

Future research should focus on refining the algorithm to increase the label accuracy while maintaining the modality-free nature and cost-effectiveness of the tool. Integrating the proposed tool with other segmentation techniques and evaluating its performance on diverse medical imaging datasets can verify its efficacy and broaden its applicability. In addition, developing a more advanced network that leverages the characteristics of the datasets created by our tool, such as the error variance, is crucial for achieving more precise recognition.

Moreover, the development of techniques for constructing personalized musculoskeletal models based on the shapes recognized by networks is vital. This is because the diagnosis of motor functions and recommendations for physical training programs rely on not only recognized shapes but also the ability of the musculoskeletal model to simulate individuals’ movements. A pipeline was developed to create personalized skeletal models from automatically recognized bone geometries^33^ using OpenSim^34^. Efforts are also being made to create finite-element muscle models^35^ and fiber models^36^ from the three-dimensional muscle geometries. In the future, rather than developing recognition technologies and model construction techniques separately, it will be necessary to develop them considering that both should function complementarily.

## Conclusions

We addressed the challenges of medical image annotation — particularly in musculoskeletal structures — by developing an innovative tool that allows non-experts to efficiently generate accurate labels. Our results indicated the effectiveness of the proposed system for reducing the time and cost associated with the annotation process while maintaining adequate quality for deep learning-based automatic segmentation. Furthermore, the versatility and adaptability of our system across different imaging modalities and anatomical structures were demonstrated, highlighting its potential to contribute to the advancement of medical image analysis. By providing a practical solution to the challenges of annotation, our research can support the development of systems for evaluating individual musculoskeletal functions by constructing subject-specific musculoskeletal models from medical images.

## Data Availability

The data that support the findings of this study are available from the corresponding author on reasonable request.

## Appendix

For guidance on how to use the Seg Muscle, please refer to the manual and video on the following website. (URL: https://sites.google.com/view/segmuscle).
